# A *TYK2* Gene Mutation c.2395G>A Leads to TYK2 Deficiency: A Case Report and Literature Review

**DOI:** 10.3389/fped.2020.00253

**Published:** 2020-05-27

**Authors:** Peilin Wu, Suqing Chen, Bin Wu, Junhong Chen, Ge Lv

**Affiliations:** ^1^The Pediatric Department, The First Affiliated Hospital of Fujian Medical University, Fuzhou, China; ^2^Chongqing Key Laboratory of Child Infection and Immunity, Children's Hospital of Chongqing Medical University, Chongqing, China

**Keywords:** *TYK2* deficiency, primary immunodeficiency, hyperimmunoglobulin E syndrome, BCG, mutation

## Abstract

Tyrosine kinase 2 (*TYK2*) deficiency was formerly defined in patients suffering from autosomal recessive hyperimmunoglobulin E syndrome (AR-HIES). In recent years, it was proposed that human *TYK2* deficiency is probably not a common cause of the AR-HIES but a distinctive illness object. In the current work, a recessive *TYK2* deficiency is reported in a patient suffering from BCG disease and recurrent respiratory infection. It was implied that this patient carried novel missense homozygous mutation (c.2395G>A, p. G799R) in the *TYK2*. Both the *in vivo* and *in vitro* experiments indicated the inhibition effects of the c.2395G>A homozygous mutation on the *TYK2* gene and protein expression. By literature review, we summarized the clinical manifestations, gene mutations, and related cytokine responses of formerly reported patients possessing *TYK2* deficiency. The core manifestation of these patients is infected by intracellular pathogens, such as mycobacteria and/or viruses. Therefore, the possibility of *TYK2* deficiency should be considered when a patient has repeated intracellular bacteria (including tuberculosis bacillus infection), repeated viral infection or eczema.

## Background

Tyrosine kinase 2 (*TYK2*), a member of the Janus kinase (JAK) family, is associated with the receptors of type I interferon (IFN), interleukin (IL)-6, IL-10, IL-12, and IL-23, has a key role in the signal transduction of these cytokines, most prominently IFNα/β (1, 2). IFN and some other cytokines, which are the critical roles in multiple adaptive and innate immune responses, transduce signals through the JAK-STAT path. Type I interferons signal through IFNAR1 and IFNAR2, which, respectively, associate with *TYK2* and *JAK1*.When the cytokines bind and induce the dimerization of their receptors, receptor-associated JAKs become phosphorylated and activated. Subsequently, the activated JAKs can phosphorylate the downstream substrates, the signal transducers and activators of transcription (STAT) molecules, then dimerizing and translocating to the nucleus for activating the particular genes transcription (3, 4). Minegishi et al. reported the first *TYK2*-deficient patient (P1) in 2006. The patient was Japanese with the triad of HIES signs (5). Interestingly, unlike P1, the HIES features were not displayed by the other 7 *TYK2*-deficient patients (P2-P8) recently recognized by Kreins et al. (1). No high serum IgE concentration, atopy, nor staphylococcal disease observed in them. They exhibited intracellular bacteria and/or viral infections, and the most typical feature is BCG disease. Therefore, it was proposed that human *TYK2* deficiency is a distinctive PID entity clinically and different from the formerly identified patients with AR-HIES (6). According to the categorization reported by the IUIS, International Union of Immunological Societies, *TYK2* deficiency was classified into Mendelian Susceptibility to Mycobacterial Disease (MSMD) (7, 8).

In this study, we report a novel *TYK2* gene mutation c.2395G>A in a Chinese patient with BCG disease using whole-exome sequencing analysis. We further summarized the clinical manifestations, gene mutations, and related cytokine responses of all reported patients with *TYK2* deficiency to review the knowledge about *TYK2* deficiency. This study was performed according to the Declaration of Helsinki (1975) with approval from the local ethics committee (ID: MRCTA, ECFAH of FMU [2019]218) of the first affiliated hospital of Fujian medical university.

## Case Presentation

### History

The patient was a boy at the age of 1 year and 11 months, the second child in the family (parents are young and nonconsanguineous), who was born in 2016 and vaccinated with BCG vaccine on the third day after birth. He was hospitalized in our hospital for bacterial pneumonia. After intradermal injection of BCG, repeated abscess and ulceration occurred at the injection site and gradually healed at 10 months. At the age of 14 months, he was hospitalized with enlarged left axillary lymph nodes. The diagnosis of BCG associated lymph node tuberculosis was made with positive staining for acid-fast bacilli and isolated Mycobacterium Bovis BCG from the discharging axillary sinuses. Chest X-ray showed no lung involvement. After the drainage of lymph node and external application of Chinese herbal medicine, the regional lymphadenopathy regressed to normal. He also suffered from recurrent respiratory tract infections (had pneumonia or upper respiratory tract infection every 1–2 months) and diarrhea since the age of 6 months (sensitive to the food firstly contacted). Pathogens found during multiple hospitalizations included Salmonella, Mycoplasma pneumonia, and Mycobacterium Bovis BCG. No considerable viral or fungal infections have happened so far. No high serum IgE concentration, atopy, staphylococcal illness, or lymphopenia was found for him. He started to say some simple words at the age of 3 years and 4 months with normal motor development. Physical examination revealed a small head circumference. His mother's brother and sister coughed and repeatedly wheezed in childhood.

### Immunologic Assessments

These data were collected when the patient was 1 year and 11 months old. The absolute eosinophil count and lymphocyte count were normal. Immunoglobin: serum IgG, IgA, IgE, and IgM levels were normal. Lymphocyte classification: the percentage of CD3^+^T cells, CD3^+^CD4^+^CD8^−^T cells, CD3^+^CD8^+^CD4^−^T cells were within the range of normal, whereas the percentage of CD45^−^CD19 ^+^ cells were higher than the normal range, and CD3^−^CD16 /CD56^+^ cells were lower (Table 1).

**Table 1:** Immunologic parameters of our patient.

**Immunologic parameters**	**Patient**	**Normal value**
Absolute lymphocyte count,/mm^3^	5,550	800–4,000
Absolute eosinophil count, /mm^3^	270	20–500
IgG, g/L	3.43	3–10
IgM, g/L	1.94	0.45–2.39
IgA, g/L	0.23	0.14–1.08
IgE, IU/ml	27.4	≤ 49
T cells, CD3^+^, %	63.2	53.88–72.87
B cells, CD19^+^, %	26.6	7.21–20.90
CD4^+^ cells, %	35.9	24.08–42.52
CD8^+^ cells, %	19.3	19.00–32.51
Natural Killer cells, CD3^−^CD16 /CD56^+^, %	3.6	13.23–26.39

### Pathogens of Respiratory Tract Infection

Data was collected after the patient was hospitalized. The results showed positive - IgM antibodies to the influenza A & B virus. which suggested the current or recent infection. IgM antibodies to m. pneumoniae and m. pneumoniae antibodies are positive (1: 160), which revealed the existence of mycoplasma pneumonia (Table 2).

**Table 2:** Pathogen detection results.

**IgM-type antibodies**	**Patient**	**Normal value**
IgM to respiratory syncytial virus	negative (-)	negative (-)
IgM to adenovirus	negative (–)	negative (–)
IgM to influenza A virus	positive (+)	negative (–)
IgM to influenza B virus	positive (+)	negative (–)
IgM to parainfluenza virus	negative (–)	negative (–)
IgM to M. pneumoniae	positive (+)	negative (–)
IgM to M. tuberculosis	negative (–)	negative (–)
IgM to legionella pneumophila	negative (–)	negative (–)
M. pneumoniae antibody (mp ab)	1: 160	1: 40

### Gene Mutation Screening

**Figure 1:**
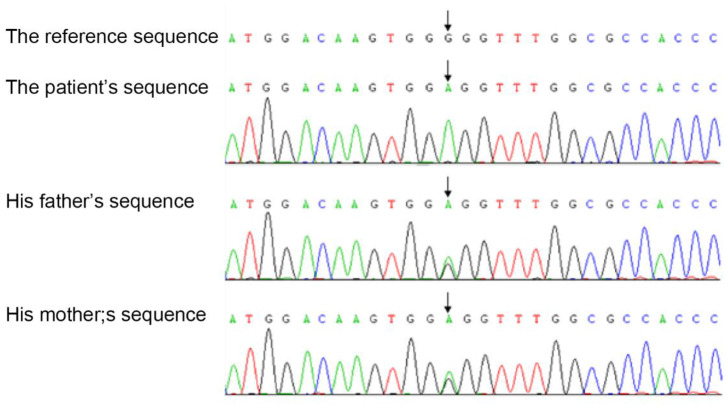
A homozygous missense mutation in exon 17 of the *TYK2* gene in our patient.

We investigated the *TYK2* gene of the members of this family except for the brother by WES (whole exon sequencing). Mutations were found in four genes, including *TYK2* (c.2395G>A, p.G799R, hom), *CFH* (c.2089C>T, p.L697F, het), *LRRC8A* (c.1250G>A, p.R417Q, het) and *NIPBL* (c.5575-27_c.5575-26delCT, –, het). The pathogenic evidences of these mutations in *CFH, LRRC8A* and *NIPBL* are insufficiency, but the possible variations of pathogenicity could not be excluded. Sequence analysis demonstrated the homozygous missense mutation in the exon 17 of the *TYK2* gene of the patient, which was heterozygous in his parents (Figure 1).

### *TYK2* Mutation (c.2395G>A) *in vivo* and *in vitro*

**Figure 2:**
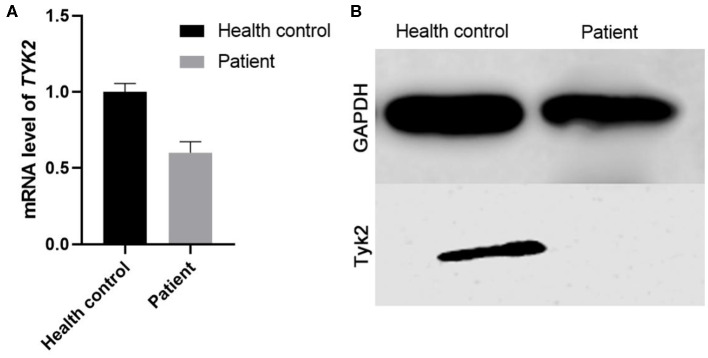
The expression of *TYK2* in these family members. **(A)** The qRT-PCR results of the patient and health control. **(B)** Western blot analysis results of Tyk2 protein in patient and health control.

**Figure 3:**
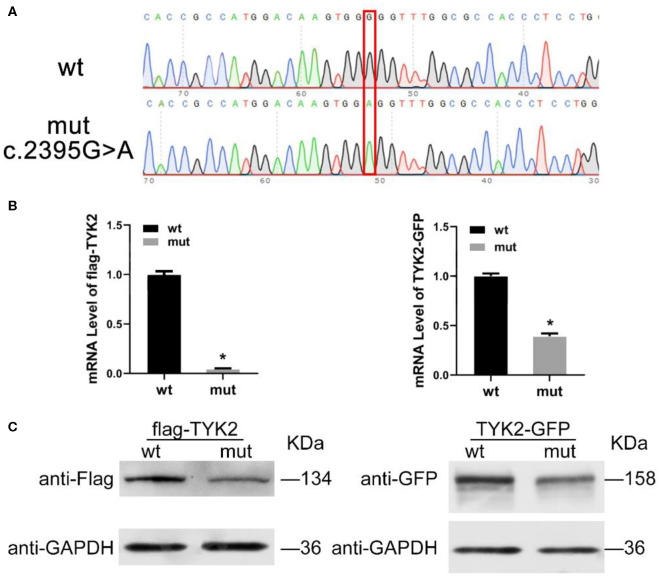
Expression of *TYK2* in wild type and c.2395G>A mutant HEK293T cell line. **(A)** The sanger analysis results of wild type and c.2395G>A mutant HEK293T cell; **(B)** mRNA expression of FLAG-TYK2 and TYK2-GFP in both wild type and c.2395G>A mutant HEK293T cell; **(C)** Western blot analysis results of FLAG-TYK2 and TYK2-GFP in both wild type and c.2395G>A mutant HEK293T cell.

qRT-PCR was carried out to detect the expression of *TYK2* in patient-derived cells with the forward primer (5′-3′ CAGATCAGACAGCACAGGGG) and the reverse primer (5′-3′ GCAGTCCTTGAAGCTGGTCT). The results showed that this mutation leads to a decrease of TYK*2* mRNA expression (Figure 2A). Besides, the western blot results showed that no TYK2 protein expression found in the patient (Figure 2B). To verify the effects of c.2395G>A on TYK2 deficiency, an *in vitro* experiment was carried out. The effects of c.2395G>A mutation on the *TYK2* gene and protein expression were revealed using two types of plasmids: p3XFLAG-CMV-7.1- TYK2 (FLAG-TYK2) and pEGFP-N1-TYK2 (TYK2-GFP). We investigated c.2395G>A (p.G799R) mutant introduced into the HEK293T cell line (Figure 3A). The qRT-PCR results showed that this c.2395G>A mutant decreased the expression of FLAG-TYK2 and TYK2-GFP (Figure 3B). To go along with this, this mutant also decreased the expressed protein of FLAG-TYK2 and TYK2-GFP in the western blot analysis (Figure 3C).

## Discussion

### Clinical Manifestations

Including our patient, there are 12 patients reported with *TYK2* deficiency (1, 2, 5, 9) (Table 3), with the average age at diagnosis of 10.8 years old. Seven patients (58%) suffered from intracellular bacterial infections. M. tuberculosis was observed in 2 cases (17%). Seven patients had recurrent virus infections (58%). Rash or atopic dermatitis eczema was observed in 2 patients (17%). Markedly increased serum IgE occurred in 3 cases (25%), and EBV-related B lymphoma and T cells lymphopenia were reported in 2 cases (17%). Different from type I high IgE syndrome caused by STAT3 mutation, none of the 12 patients had special facial features, abnormal bone development or pathological fractures, delayed deciduous tooth loss, or aneurysms.

**Table 3:** Information about reported TYK2-deficient patients.

	**First author**	**Sex**	**Country**	**Age**	**Atopy/** **dermatitis**	**High IgE levels**	**S. aureus infection**	**Viral infection**	**Intracellular bacterial infection**	**Fungal infection**	**BCG vaccination**	**Gene mutation**	**Protein mutation**
P1	Minegishi	M	Japan	22	yes	yes	yes	HSV, PI3, MC	BCG *Salmonella*	C. albicans	yes	Exon 4 c.550_553delGCTT	C70HfsX21
P2	Kreins	M	Turkey	17	no	no	no	VZV	BCG, *Brucella*	no	yes	Exon 16 c.2303_2311del	L767X
P3	Kreins	F	Morocco	14	no	no	no	no	*M. tuberculosis*	no	yes	Exon23 c.3318_3319insC	T1106HfsX4
P4	Kreins	M	Morocco	14	no	no	no	unknown	unknown	no	yes	Exon23 c.3318_3319insC	T1106HfsX4
P5	Kreins	M	Iran	5	no	no	no	no	BCG	no	yes	Exon5 c.462G>T	E154X
P6	Kreins	F	Iran	2	no	no	no	yes	BCG	no	yes	Exon5 c.462G>T	E154X
P7	Kreins	F	Iran	9	no	no	no	no	*M. tuberculosis*	no	yes	Exon3 c.149delC	S50HfsX1
P8	Kreins	M	Argentina	11	no	no	no	HSV	No	no	yes	Exon13 c.1912 C>T	R638X
P9	Fuchs	M	Kurdish	5	yes	yes	yes	no	No	no	no	Exon7 c.647delC	P216RfsX14
P10	Nemoto	M	unknown	15	no	no	no	EBV-associated B-cell lymphoma, varicella	No	no	yes	compound heterozygous mutations c.209_212 del GCTT /c.691 C > T	C70SfsX21/R231W
P11	Nemoto	F	unknown	14	no	no	no	EBV-associated B-cell lymphoma, Varicella	Unknown	no	yes	compound heterozygous mutations c.209_212 del GCTT /c.691 C > T	C70SfsX21/R231W
P12	Peilin	M	China	2	no	no	no	Influenza virus	MP, BCG	no	yes	Exon17 c.2395G>A	G799R
Total		M:F = 8:4		10.8	2/12 (0.17)	2/12 (0.17)	2/12 (0.17)	7/12 (0.58)	7/12 (0.58)	1/12 (0.08)	11/12 (0.92)		

*BCG, Bacillus Calmette – Guerin; MP, mycoplasma pneumonia*.

### Pathogenesis

The mammalian JAK family has four members: JAK1, JAK2, JAK3 and tyrosine kinase 2 (TYK2). Tyk2 as an essential element, which is activated by an array of cytokine receptors in IL-12 and type I-IFN signaling. A mutation in this gene has been associated with HIES (5, 10). In this case, c.2395G>A is a missense mutation that causes the amino acid to change from G to R, which may restrict the function of TYK2 protein. Our results also support this inference, and we did observe decreases in gene expression and protein levels in patient-derived cells and HEK293T cells. Although HEK293T cells have lower background factors, they still cannot fully simulate patient-derived cells, so the results of the two cells are somewhat different, but at least the trends of both cell types are consistent. For its pathogenic mechanism, viral and/or mycobacterial infections are the main clinical phenotypes of *TYK2* deficiency, initiated by diminished reactions to IL-12 and IFN-α/β. A key cytokine in generating and regulating the cellular immune response needed for the elimination of intracellular pathogens from macrophages is IFN-γ. *TYK2* mediates signal transduction between cytokine receptors and STATs. Both IL-12 and IL-23 can promote the production of IFN-γ. *TYK2* mutation weakened the response of NK and T cells to IL-12 and IL-23, resulting in insufficient IFN-γ creation (11, 12). When cells cannot secrete normal levels of IFN-γ, they are vulnerable to poorly pathogenic mycobacteria, and develop into MSMD (12). In addition, the main factor of the innate antiviral defense of all cells is represented by IFN-α/β (13). The largely impaired or abolished of IFN-α/β responses probably contribute to the incremented sensitivity to viral infections. However, virus control via type-III IFN (IFN-λ) signals could be a compensatory role contributing to the mild phenotype in *TYK2* deficient patients regardless of an intense decrease in IFN-α/β mediated antiviral activity (14). Nemoto reported a recessive partial *TYK2* deficiency in two siblings who presented with T-cell lymphopenia characterized by low naïve CD4+ T-cell counts and who developed EBV-associated B-cell lymphoma (2). *TYK2*-deficient patients presented with infections associated with the herpesvirus family (1), thus, *TYK2*-deficient patients might be susceptible to EBV (15). Correspondingly, it was observed that not all *TYK2*-deficient patients had poor control of the virus. Among these informative patients, no incremented susceptibility to viral infections was found nearly in half of them. Very mild candidiasis was found in P1 (restricted to a small number of episodes of slight oral candidiasis), not in P2-P11, and P12. The normal circulating IL17 T cells probably explained their apparent lack of CMC. The response to IL-23 is weakened but not stopped in *TYK2*-deficient patients. The susceptibility of the patient to extracellular bacteria might be described at least partly by the deficiency in IL-23 signaling. The high quantity of serum IgE and the atopic dermatitis-like skin inflammation in the patient could be caused by the hastened Th2 differentiation. *TYK2* mutation probably promotes Th2 cell differentiation, leading to incremented producing of IL-4, IL-5, and IL-13, associated with atopic dermatitis and eczema. Besides, different mutation types may have different impact on the expression of *TYK2*, which lead to different patient characterization. Sequencing results implied that our patient carried novel missense homozygous mutation (c.2395G> A, p. G799R) in the *TYK2*. As a supplement, this mutation type was is included in the genome Aggregation Database (gnomAD), suggesting that this was not a private mutation. The CADD_score, GDI and MSC-SIFT_Score were A:25.6, 1605.550 and 0.243, respectively. Patients comes from consanguineous family might have other defects in addition to the TYK2 deficiency, which might influence the clinical phenotype.

### Differential Diagnosis

All the 12 reported *TYK2*-deficient patients were infected by potential infection source (S. aureus infection/Viral infection/Intracellular bacterial infection/Fungal infection), which might be one of the important indicators for diagnosis. In addition, high serum IgE concentration is not universal in *TYK2*-deficient patients. In our *TYK2* deficiency case, he was sensitive to the food firstly contacted, which indicated the intolerance to bacteria. Also, the family history of childhood wheezing cannot be ignored. Combined with the reported *TYK2* deficiency cases, we summary the main features of *TYK2* deficiency: (a) *TYK2* gene mutation; (b) Autosomal recessive inheritance; (c) The number of lymphocytes is normal, and multiple cytokine signaling defect; (d) Susceptibility to viruses or intracellular bacteria; (e) With/without elevated IgE. Therefore, the possibility of *TYK2* deficiency should be considered when a patient has repeated intracellular bacteria (including tuberculosis bacillus infection), repeated viral infection or eczema, especially with a family history. An accurate genetic diagnosis could indicate susceptible pathogens conducive to accurate treatment.

## Data Availability Statement

All datasets made for this work are provided in the article/supplementary material.

## Ethics Statement

This study was performed according to the Declaration of Helsinki (1975) with an approval from the local ethics committee (ID: MRCTA, ECFAH of FMU [2019]218) of the first affiliated hospital of Fujian medical university. Written informed consent was obtained from the parents of the participant for the publication of this case report.

## Author Contributions

PW, JC, and SC contributed to the literature searching. PW transcribed the first draft of the manuscript. SC and BW contributed to the modification of the discussion section in the manuscript. GL contributed to Western Blot verification experiment. All writers helped manuscript revision, read and approve the acquiesced version.

## Conflict of Interest

The authors declare that the research was conducted in the absence of any commercial or financial relationships that could be construed as a potential conflict of interest.
